# Extracting geriatric syndromes from electronic health records: a scoping review

**DOI:** 10.1007/s41999-025-01388-5

**Published:** 2026-01-09

**Authors:** C. Squires, A. Duignan, A. Peterson, A. H. M. Kilgour, T. C. Russ, S. R. Cox, S. D. Shenkin

**Affiliations:** 1 Ageing and Health Research Group, and Advanced Care Research Centre, Usher Institute, University of Edinburgh, 5 Little France Rd, Edinburgh, EH16 4UX UK; 2 NHS Lothian, Edinburgh, Scotland, UK; 3 Alzheimer Scotland Dementia Research Centre, School of Philosophy, Psychology, and Language Sciences, University of Edinburgh, 7 George Square, Edinburgh, EH8 9JZ Scotland, UK; 4 Lothian Birth Cohorts, Department of Psychology, University of Edinburgh, 7 George Square, Edinburgh, EH8 9JZ Scotland, UK; 5 Lothian Birth Cohorts, Edinburgh Futures Institute, University of Edinburgh, 1 Lauriston Place, Edinburgh, EH2 9EF Scotland, UK

**Keywords:** Geriatric syndromes, Electronic health records, Clinical coding, Multifactorial

## Abstract

**Introduction:**

Geriatric syndromes are multifactorial ageing-related presentations. There is limited consensus regarding which presentations should be considered geriatric syndromes. Clinical codes generated following healthcare contacts are commonly used to identify diverse medical conditions. It is well demonstrated that geriatric syndromes are harder to identify using codes, possibly partly due to varying definitions, although identifying information is often located in unstructured text. Accurately identifying geriatric syndromes in healthcare records may therefore require different approaches compared to more firmly defined conditions. We review how studies using Electronic Health Records (EHRs) and derived databases align or differ in terms of geriatric syndromes selected for study, and how the syndromes are identified in the data sources.

**Methods:**

The review was registered on the Open Science Framework (https://osf.io/9ejhq). Databases were searched in October 2024. Of 8086 titles screened, 74 studies had full text review, and 12 studies were included.

**Results:**

Twelve studies from 7 countries identified 31 geriatric syndromes from primary databases (*N* = 6) or secondary databases (*N* = 6) using diagnostic codes, screening tools, or free text. The five most common geriatric syndromes were falls, functional decline, incontinence, delirium and malnutrition. Thirteen geriatric syndromes were included in two or more studies. Eight studies used more than one identification method. One study used two methods for all included syndromes.

**Key conclusions:**

There is heterogeneity in what presentations studies include as geriatric syndromes, and how they identify them in EHRs. It is essential that researchers clearly describe their included geriatric syndromes, reason for selection, and identification methods.

**Supplementary Information:**

The online version contains supplementary material available at 10.1007/s41999-025-01388-5.

## Key summary points

**Aim:**

This scoping review investigates how studies using electronic health records or derived databases align or differ in their choices of geriatric syndromes, and the methods used to identify included geriatric syndromes in the data sources.

**Findings:**

Twelve studies identified 31 geriatric syndromes, the most common being falls, incontinence, delirium, functional decline and malnutrition. There was variation in both choices of syndromes, definitions of individual syndromes, and methods of identifying them in the data sources across included studies.

**Message:**

There is heterogeneity in both choice of geriatric syndromes and identification methods, and this may hinder opportunities for cross-study comparability.

**Supplementary Information:**

The online version contains supplementary material available at 10.1007/s41999-025-01388-5.

## Introduction

Geriatric syndromes, sometimes referred to as frailty syndromes, have been defined as ageing-associated presentations with causative roots in several organ systems (i.e. are multifactorial), which can be destabilised by stressors. They are considered final common pathways of complex pathophysiological processes [1]. Early studies and reviews suggested a key characteristic is the tendency to share risk factors, including age, cognition, reduced mobility and functional impairment [2, 3]. Homeostatic decompensation has also been proposed as a unifying mechanism [4].

Consensus regarding which clinical presentations should be described as geriatric syndromes has proved elusive. Several national Geriatric Medicine societies have formulated lists in different contexts [5–7], with falls, delirium, incontinence and immobility consistently included across them. These presentations mirror the historic ‘geriatric giants’ described by Bernard Isaacs in 1965 [8] and also partially align to the modern ‘5Ms’ concept [9, 10]. Other conditions described as geriatric syndromes include malnutrition, dysphagia, depression, pressure ulcers, sensory impairments and sociocultural factors, amongst many others, some of which might not be accurately described as syndromes. Frailty is described as a geriatric syndrome, though it might also be considered a modifier as it is associated with the development of other geriatric syndromes, and accentuates outcomes when combined with other syndromes [11, 12]. Many geriatric syndromes are associated with important outcomes including admission to hospital [12, 13], institutionalisation [13, 14], high healthcare related costs [13], depression [15, 16], quality of life [17], and mortality [18]. Notably, geriatric syndromes can sometimes be stronger predictors of outcomes than more readily defined or single-organ conditions [19].

Challenges in defining geriatric syndromes may contribute to underdiagnosis and less consistent coding in clinical records than ‘standard medical conditions’ [20, 21]. Downstream issues may also be coded preferentially, for instance recording a fracture, rather than the fall causing it. Coding rates of some geriatric syndromes have changed with time; whilst delirium coding has recently increased [22], functional dependence remains poorly coded, and incontinence coding varies significantly between counties [23]. Some of this may reflect a previous tendency to deprioritise these syndromes, as well as the availability of suitable codes.

Identification of geriatric syndromes in clinical records may be increased by searching free or unstructured text, such as discharge letters and clinical notes, compared to using clinical codes (such as ICD-10) alone [24, 25]. Some syndromes can also be identified via documented screening tools, e.g. the 4AT or CAM (delirium). These different identification methods may particularly benefit syndromes less amendable to traditional coding or which are otherwise challenging to identify, e.g. walking difficulty [26, 27].

Electronic Health Records (EHRs) are vital for clinical practice but also are rich research resources. EHRs usually cover a defined healthcare provider or service and may incorporate secondary databases covering specific populations or insurance programmes. Some EHRs have been designed with a research focus, e.g. MIMIC-IV [28]. Many countries now have widespread uptake of EHRs. US and Australia have demonstrated uptakes of 96 and 97%, respectively, as of 2021, and Türkiye has similarly demonstrated increased uptake from 97 to 100% between 2017 and 2021 [29–32]. There can be large variation in the scope of clinical information held in different EHRs meaning direct comparison of available data may prove challenging [28–31].

Determining which geriatric syndromes are included in EHR-based studies and which methods are used to identify them may enable harmonisation of future approaches to these conditions. This could increase the feasibility of cross-study synthesis and improve statistical powering for future meta-analytic frameworks. It will also benefit researchers using routinely collected healthcare data to explore predictors and outcomes relevant to older people.

This scoping review aims to describe the range of conditions described as geriatric syndromes in published studies, and the methods used to identify them in EHRs.

## Methods

### Protocol and registration

The protocol was registered retrospectively on the Open Science Framework website on 28/03/25 (10.17605/OSF.IO/9EJHQ). Reporting follows the PRISMA-ScR (Preferred Reporting Items for Systematic Reviews and Meta-Analysis extension for Scoping Reviews) checklist [33] (see Appendix 1).

### Eligibility criteria

Full eligibility criteria available in Appendix 2.

Inclusion criteria.

- Included older adults
- Referred to included conditions as ‘geriatric syndrome’ or accepted synonyms.
- Included at least two geriatric syndromes.
- Used electronic health records (EHR), or her-derived datasets, as data source.

Exclusion criteria.

- Full text not available in English
- Identification method unclear for all included syndromes
- NLP-based studies

### Information sources

PubMed, CINAHL Plus and SCOPUS databases were searched from inception. Grey literature sources including national geriatric society resources were also searched.

### Search strategy and source selection

A systematic search was formulated after discussion with medical librarian MD. This combines concepts of geriatric syndromes and synonyms, and electronic health records and synonyms (full search strategy Appendix 3).

Search results were imported into the Covidence software management system (Veritas Health Innovation) for de-duplication and screening. Titles were independently screened by CS and either AD or AP. Discrepancies were resolved by discussion and senior reviewer opinion (SDS) where required. Identified studies underwent full text review. References of included studies were searched.

### Data charting

A data extraction table was designed by CS. Identification methods were separated into ‘clinical codes’, ‘screening tools’ (‘tools’), ‘free text’, and ‘unclear’. The free text category included methods specifically referencing this or strongly indicative descriptions, e.g. ‘taken from nursing notes’. Choice of method and number of methods used in individual studies was recorded, including whether studies used single, or multiple methods for included syndromes, and differing methods between syndromes. For each syndrome included in two or more studies, all identification methods were recorded, with percentages for each category. Comparative statistics were not performed given the sample size and study heterogeneity.

### Critical appraisal of sources

We anticipated a thematic overview due to the expected heterogeneity in geriatric syndrome selection and methodology. We used the CASP checklist [34] to support data extraction, which was categorised under the following headings: data sources, geriatric syndrome synonym use, syndromes included individually and across studies, and method of syndrome identification individually and across studies (see Appendix 4).

## Results

### Characteristics of included studies

**Fig. 1:**
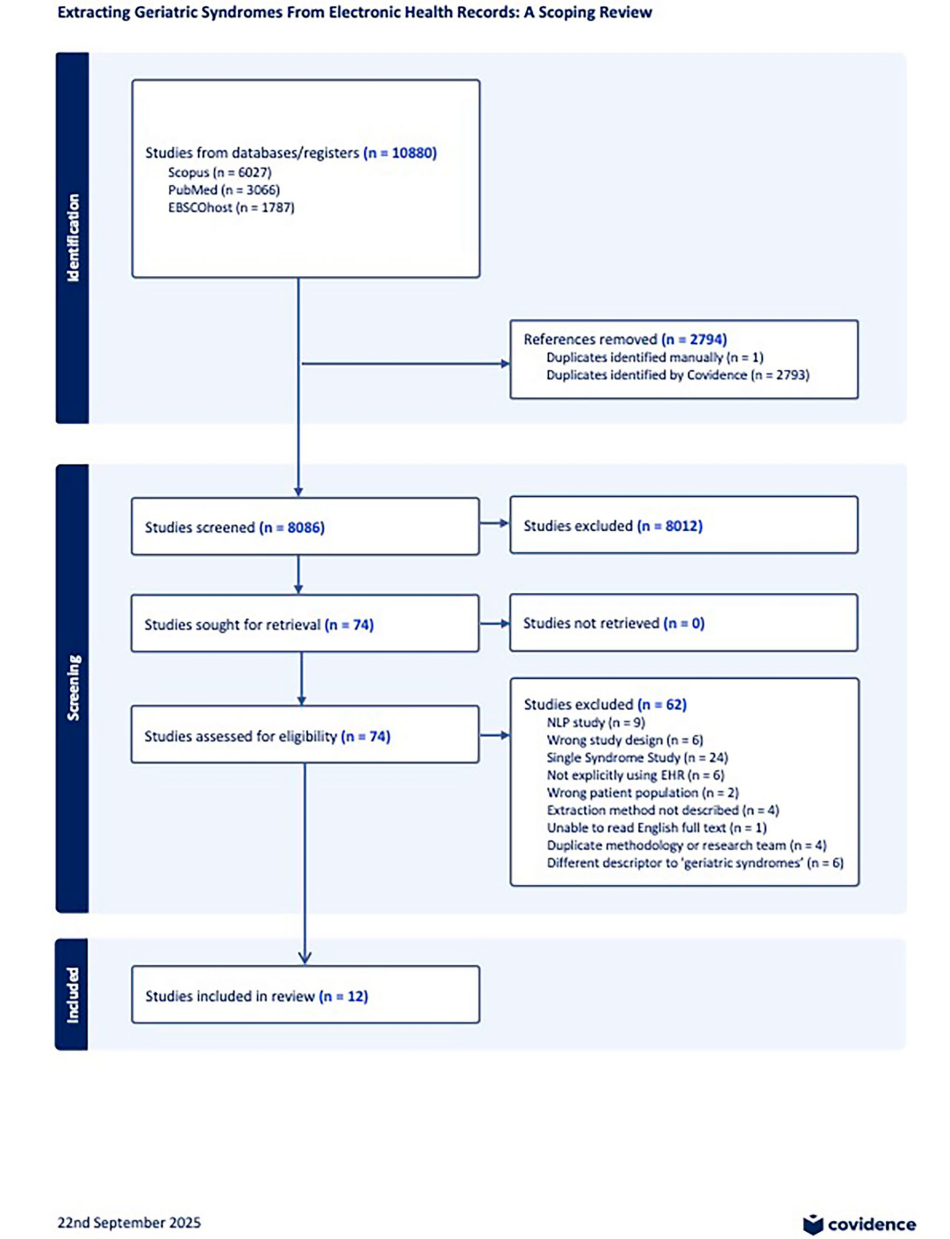
Selection of sources

Of 8086, 74 studies had full text review. 12 studies, from 7 countries, published between 2010 and 2024, were included. As this review focusses on identification methods used in studies rather than specific outcomes, where multiple studies from the same first author used identical syndromes and methods, we included a single study, choosing either the most recent, or most methodologically comprehensive (see Fig. 1).

Six studies (50%) extracted data directly from electronic health records [35–40]. One study [36] did not specify using an EHR, but information in the paper suggested this was likely. Six studies used secondary databases: Hospital Episode Statistics (England) [41, 42]; MIMIC-14 (Medical Information Mart for Intensive Care IV) (US) [41], CRPD (Clinical Research Practice Datalink) (England) [43], THL (Finnish Institute for Health and Welfare), and FHDR (Finnish Hospital Discharge Register) (Finland) [44], and ‘bespoke with deidentified EHR data’ [11]. Nine studies used the term ‘geriatric syndrome’, two used ‘geriatric characteristic’ and one used ‘frailty syndrome’. Eight studies (66%) had an identifiable geriatrician author [11, 35–40, 44]. The four largest studies were dataset derived and used codes to identify geriatric syndromes [42, 43, 45, 46]. See Table 1.

**Table 1:** Characteristics of included studies

First author	Title	Year	Country of study	Data source	Population size	Age range (years)	Syndromes included	Extraction methods	Geriatrician authorship
Chew [11]	Frailty screening and detection of geriatric syndromes in acute inpatient care: impact on hospital length of stay and 30-day readmissions	2023	Singapore	Secondary database	773	65+	Bladder or bowel incontinence	Tool	Yes
							Cognitive impairment	Tool	
							Functional decline	Multiple methods (tool/free text)	
							Recurrent falls	Tool	
							Malnutrition	Tool	
							Poor oral health	Tool	
De Bruecker [35]	Could geriatric characteristics explain the under-prescription of anticoagulation therapy for older patients admitted with atrial fibrillation?	2010	Belgium	Electronic health records	768	71–95, mean 84 (SD 5)	Cognitive disorders	Unclear	Yes
							Depression	Tool	
							Falls	Free text	
							Functional dependence	Tool	
							Incontinence	Unclear	
							Malnutrition	Tool	
Faitna [45]	Has multimorbidity and frailty in adult hospital admissions changed over the last 15 years? A retrospective study of 107 million admissions in England	2024	England	Secondary database	107,000,000 patient spells	65 + (whole cohort stratified into 18-44, 45-64, 65+)	Anxiety and depression	Codes	No
							Delirium	Codes	
							Dementia	Codes	
							Dependence and care	Codes	
							Falls and fractures	Codes	
							Incontinence	Codes	
							Mobility problems	Codes	
							Pressure ulcer	Codes	
							Senility	Codes	
Liang [41]	Elderly patients with dysphagia in the intensive care unit: Association between malnutrition and delirium	2024	USA	Secondary database	2273	65+	Delirium	Tool	No
							Malnutrition	Codes	
Lin [36]	Burden and impact of multifactorial geriatric syndromes in allogeneic hematopoietic cell transplantation for older adults	2019	USA	Presumed Electronic health record	527	60+	Delirium	Multiple methods (codes/free text)	Yes
							Falls	Multiple methods (codes/free text)	
Lozano-Montoya [37]	Mortality risk factors in a Spanish cohort of oldest-old patients hospitalized with COVID-19 in an acute geriatric unit: the OCTA-COVID study	2021	Spain	Electronic health record	300	75+	Delirium	Tool	Yes
							Dementia	Tool	
							Functional status	Tool	
							Polypharmacy	Free text	
Melzer [43]	Much more medicine for the oldest old: trends in UK electronic clinical records	2015	England	Secondary database	27,109	85+	Dizziness (including vertigo and syncope)	Codes	No
							Falls	Codes	
							Fractures	Codes	
							Incontinence (urinary and faecal)	Codes	
							Skin ulcers (including bed sores)	Codes	
Ohuabunwa [38]	Clinical presentation of COVID‐19 and association with outcomes among hospitalised older adults	2023	USA	Electronic health record	134	65+	Altered mental status	Free text	Yes
							Falls	Unclear	
							Weakness	Unclear	
Oud [39]	Interaction between geriatric syndromes in predicting three months mortality risk	2022	Netherlands	Electronic health record	4478	70+	Delirium risk	Free text	Yes
							Falls risk	Free text	
							Malnutrition	Tool	
							Physical Impairment	Tool	
Rönneikkö [44]	Reasons for home care clients’ unplanned Hospital admissions and their associations with patient characteristics	2018	Finland	Secondary database	6812	63+	Anorexia	Codes	Yes
							Cachexia	Codes	
							Other symptoms and signs involving cognitive functions and awareness	Codes	
							Dizziness and giddiness	Codes	
							Other symptoms and signs involving general sensations and perceptions	Codes	
							Dry mouth, not otherwise specified	Codes	
							Dyspnoea	Codes	
							Faecal incontinence	Codes	
							Falls	Codes	
							Idiopathic hypotension	Codes	
							Malaise and fatigue	Codes	
							Malnutrition	Codes	
							other nutritional deficiencies	Codes	
							Sequelae of malnutrition and other nutritional deficiencies		
							Syncope and collapse	Codes	
							Urinary incontinence (stress)	Codes	
							Urinary incontinence (NOS)	Codes	
							Volume depletion and disorders of fluid, electrolyte and acid–base disturbance	Codes	
							Abnormal weight loss	Codes	
Soong [42]	Quantifying the prevalence of frailty in English hospitals	2015	England	Hospital Episode Statistics database	50,540,141 patient spells	65+	Delirium	Codes	No
							Dementia	Codes	
							Falls and fractures	Codes	
							Functional dependence	Codes	
							Incontinence	Codes	
							Mobility problems	Codes	
							Pressure ulcers	Codes	
							Anxiety/depression		
							Senility	Codes	
Szklarzewska [40]	A comparison of clinical characteristics between old and oldest-old patients hospitalised for SARS-COV2	2023	Belgium	Electronic health record	986	75+ (stratified to 75+ and 85+)	ADL assistance	Tool	Yes
						-	Cognitive impairment	Unclear	
							Depression	Unclear	
							Falls	Free text	
							Frailty	Tool	
							Incontinence	Unclear	
							Malnutrition	Multiple methods (tool/free text)	
							Swallowing disorders	Unclear	
							Number of medications taken at home	Unclear	
							Hospitalisation in last 6 months	Unclear	

### Summary of included syndromes

**Fig. 2:**
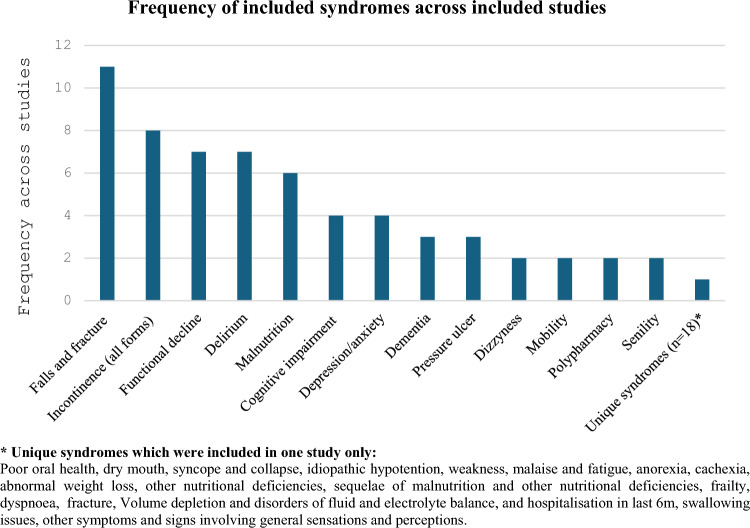
Frequency of included geriatric syndromes across included studies

31 geriatric syndromes were described across all studies, with 13 included in two or more studies. The most frequent were falls, incontinence, functional decline, delirium, and malnutrition (see Fig. 2), which all featured in four or more studies. 18 syndromes featured only once across studies. These were collated into a ‘unique syndrome group’ and thematically subdivided. One study included frailty as a geriatric syndrome [40]. Two studies included frailty separately to their described geriatric syndromes [11, 37]. It was therefore categorised as a unique syndrome.

### Defining geriatric syndromes: combining similar terms

Equivalent presentations described using different synonyms were combined where appropriate. For example, ‘functional decline’, ‘functional dependence’ and ‘dependence and care’ were combined into the category of ‘functional decline’ based on author definitions and descriptions. Similarly, non-specific ‘cognitive impairment’ and similar terms were collated as ‘cognitive impairment’. Clearly defined dementia and delirium were categorised separately. ‘Altered mental state’ was combined with delirium based on description.

The ‘incontinence’ category combined specified urinary incontinence, specified faecal incontinence, combined urinary and/or faecal incontinence, and undetermined incontinence. Four of the seven studies including incontinence combined urinary and faecal types together, and one study clearly separated them. The remaining two studies did not clearly describe subtypes.

Two studies combined ‘falls and fracture’ into a single category [42, 45]. For this review, these two studies were incorporated into the falls category. One study included fracture separately from falls, so fracture was also included as a unique syndrome.

Seven studies included malnutrition as a geriatric syndrome. One study included malnutrition alongside several adjacent concepts, e.g. cachexia [46]. These were handled separately as unique syndromes. One study population comprised of patients with dysphagia; this was therefore not counted as an included geriatric syndrome [41].

### Frequency of individual geriatric syndromes across studies

There were 13 geriatric syndromes included at least twice across all studies. The five most frequent syndromes were falls (11 studies), incontinence (8), functional decline (7), delirium (7) and malnutrition (6). Two studies included both mobility problems and functional dependence. Functional dependence was collated into the functional decline category. ‘Mobility problems’ was judged to be a distinct presentation from functional decline, so was included separately.

The 18 unique syndromes (from five studies) aligned to five themes: oral health (two syndromes), causes of collapses and falls (two syndromes), energy intake and metabolism (five syndromes), malaise and fatigue-related symptoms (two syndromes), and ‘other’ (seven syndromes). 12 unique syndromes came from one study [44]. See Fig. 2.

### Choice of identification methods

Four studies used a single identification method (clinical codes) for all geriatric syndromes. Three studies used ICD-10 codes [42, 44, 45], and one used Read codes (mainly used in primary care) [43]. Eight studies incorporated more than one identification method, for example using tools for some geriatric syndromes and free text for others [39], or combinations of tools and free text [35].

**Fig. 3:**
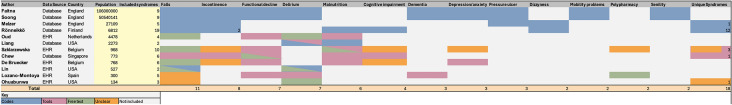
Summary of syndromes included across individual studies and their identification methods. Where two methods were used, this is indicated by split colour cells

Four studies used two or more methods (“multiple methods”) to identify single syndromes. Two used a combination of free text and codes [11, 36], and two used a combination of free text and tools [40, 41]. One study used two methods for all syndromes (noting this study included just two geriatric syndromes) [36]. Overall, seven studies used free text either in a single or combined identification method [11, 35–40]. Two studies partially described an identification method but featured enough information for reasonable assignation [39, 41]. Methods in four other studies were unclear [35, 37, 38, 40]. English studies tended to use codes alone. The study with the most frequent use of tools was from Singapore [11]. See Fig. 3.

### Identification methods of syndromes across all studies

The four most common syndromes were consistently included across studies from different countries, of different population sizes, and were identified with a variety of methods. Malnutrition was less commonly included in the largest studies, possibly due to its complex handling using clinical codes (the method used by these studies). Of the syndromes included in two or more studies, four (dizziness, mobility problems, pressure ulcers and senility) only featured in large dataset studies and were only identified using codes [42–45]. The additional nine syndromes were identified using several methods. Syndromes with several identification methods across studies included the most common (falls, incontinence) as well as rarer syndromes, e.g. depression and polypharmacy.

Across all studies, excluding unique syndromes, 12 syndromes were identified using codes, eight syndromes using screening tools, and three syndromes using standalone free text. Four syndromes were identified using multiple methods (i.e. two methods in the same study, one of which was always free text). Overall, five syndromes across studies (falls, delirium, functional decline, malnutrition, and polypharmacy) were identified using free text as either a standalone or multiple method. This suggests that free text was generally used for more commonly included syndromes. Of the 18 unique syndromes, 13 were identified using codes, two used tools and three were unclear.

### Neuropsychiatric conditions

Four studies included cognitive impairment or related terms [11, 35, 40, 44]. Four (different) studies included delirium [36, 38, 39, 41], and three studies included both delirium and dementia [37, 42, 45]. There was no noted temporal change in the choice of neuropsychiatric syndrome. Two studies did not describe the identification method for cognitive impairment [35, 40] though methods were consistently described for delirium (two codes, two free text, two tools (CAM, CAM-ICU), one multiple methods) and dementia (two codes and one tool (global deterioration scale)). *Detailed descriptions of cognitive syndrome descriptions and identification are in Appendix 5*.

### Incontinence

Seven studies included incontinence [11, 35, 40, 42–45], with one study including urinary and faecal incontinence separately [44], giving a total frequency of eight. Codes were the most frequent identification method. Four studies (mostly large and dataset-derived) used combined faecal and urinary incontinence [11, 42, 43, 45]. Two studies did not define incontinence or identification method [35, 40].

## Discussion

There is little consistency in the presentations described as geriatric syndromes or the methods used to identify them. In this scoping review of 12 studies from 7 countries, of which half used primary electronic health records and half derived datasets, we identified a total of 31 syndromes. The five most commonly identified syndromes (falls, incontinence, functional decline, delirium and malnutrition) were included in four or more studies. A further eight syndromes were included in two or more studies, with the remaining 18 (over half of all syndromes) included in a single study alone. This suggests the descriptor of ‘geriatric syndrome’ currently lacks clarity and consensus, though there was some agreement regarding the most encountered presentations.

Defining geriatric syndromes remains complex; should the definition adhere to the traditional model of multifactorial presentations which fluctuate in response to stressors and may therefore be associated with acute healthcare needs? Or should the term encompass any condition important to older adults? Presentations such as malnutrition or depression would not usually be considered classically fluctuant or firmly ageing related. Other presentations described in this review are not typically considered syndromic (e.g. pressure ulcers, polypharmacy, and hospital admission) or are rarely used in current clinical practice (e.g. senility). The differential handling of frailty further demonstrates a consensus gap.

Most syndromes in this review have been described elsewhere as geriatric syndromes. Dysphagia was infrequent in this review, though has gained recent traction as a geriatric syndrome [47–50]. Similarly, the evolving status of some conditions may be relevant. Sarcopenia (absent from included studies but present in the literature [18, 50–52]) was incorporated into ICD-10 in 2016, meaning that code-based studies prior to this would have been unable to include it [53]*.* Whilst these aspects could imply that this review does not accurately reflect the broader literature, it more likely confirms the laxity inherent within the geriatric syndrome concept. Until a clear consensus is reached, studies using geriatric syndromes will remain challenging compared to the detriment of researchers. This is further complicated by the variety of definitions and identification methods used for individual syndromes.

However, it is noteworthy that the four most included presentations (falls, incontinence, functional decline and delirium) were included across studies of varying size, overlap with key clinical geriatric medicine concepts including the geriatric giants described by Bernard Isaacs, and the more modern ‘5Ms’ framework, and feature across three published national geriatrics society lists of geriatric syndromes [5–7, 9, 52]. This suggests that despite the variation in geriatric syndromes included in this review, there is a smaller group that could be considered to be core, given their inclusion across studies of varying demographics and in key literature. Furthermore, four studies [37–40] investigating relationships between geriatric syndromes and patient outcomes in the Covid-19 pandemic all included falls and delirium. This reinforces their status as key geriatric syndromes.

Just two studies [42, 45] included ‘mobility problems’ (identified via ICD-10 codes) in this review. This is of interest as immobility features consistently across the described national society lists and conceptual frameworks. We also note that there is also the potential for debate in classing functional decline (and associated terms) as an independent geriatric syndrome, instead of as a measure of disability (in keeping with the WHO International Classification of Functioning, Disability and Health framework), despite these terms being included throughout both this review and the broader literature [54,55]. This again demonstrates the inherent complexity of geriatric syndromes as a concept and suggests a need for consensus, as well as careful consideration of what terms are selected and what is being described.

This review found heterogeneity in the handling of neuropsychiatric disorders and incontinence. Cognitive impairment was included in four studies and lacked a consistent definition, with the term potentially combining disorders (e.g. delirium, diagnosed and undiagnosed dementia) with very different trajectories. Similarly, combining modalities of incontinence is relevant given that faecal incontinence has a different pathophysiology and causes more institutionalisation than urinary incontinence alone [56, 57]. A lack of accuracy and consistency regarding how these conditions are defined is disappointing given their substantial impact. We acknowledge that specific research questions and contexts, as well as pragmatism, may have influenced decisions. Moving forward, a key recommendation for studies considering geriatric syndromes may not only be to promote the inclusion of key syndromes such as falls and delirium but also to be explicit in how all included syndromes are defined, particularly when the impact of different subtypes on patients and healthcare systems is known to differ.

### Identification aspects

Clinical coding may underserve the identification of some geriatric syndromes. Supporting evidence may often be found in free text sources, leading to increased identification yield [20, 26]. This is relevant given the impact of geriatric syndromes both on patient outcomes, but also service aspects, including healthcare costs and iatrogenic harm, meaning that accurate quantification is critical [58, 59]. Acknowledging this, we were interested to see how studies selected their identification methods. It should be noted that coding a condition often entails consideration of supporting evidence which may include tool and free text aspects.

In this review, codes were the most common identification method, despite their demonstrated limitations. Seven studies used free text, and whilst four used multiple methods for individual syndromes to an extent, only one study used two methods for all syndromes. The reasons for the choice of identification methods in studies using several methods were rarely described, and it is unclear whether this was influenced by specific attempts to optimise yield or more likely reflected accessibility and feasibility within individual study design and constraints. Pragmatically, choices of both syndromes and methods may be influenced by available options (i.e. choice of codes in different systems or choices of tools embedded and recorded in EHRs). Complete consistency is unrealistic. However, careful consideration and recording of decisions relating to choices of identification methods regarding geriatric syndromes is prudent given the established issues with under-recognition and the potential of free text sources to reduce this gap.

### Strengths

This study used broad search terms and an inclusive approach to data sources to provide a comprehensive overview of syndrome description and identification methods. The authors performing title screening are experienced geriatricians. A systematic approach was used, with senior medical librarian assistance, methodology informed by PRISMA-ScR, and critical appraisal by the CASP tool, followed by a thematic synthesis. Most studies (75%) featured geriatricians as authors and studies were produced across differing nations and healthcare systems, giving a broad overview of potential identification methods of different geriatric syndromes.

### Limitations

Relevant studies may have been missed if they did not reference ‘geriatric syndromes’ or synonyms. Our search may also have missed studies published in data-science journals. Studies were excluded if we could not accurately establish if data sources were EHR, or EHR-derived, despite non-EHR studies potentially using comparable methodology. Single condition studies were excluded partly to make screening numbers manageable and to focus on studies describing alignment to the geriatric syndrome concept; this may have impacted perceptions regarding identification methods of syndromes. Whilst NLP has shown promise in identifying many of these syndromes [60], and is becoming more embedded methodologically, these techniques may not be appropriate for all contexts. For this reason, we excluded studies using this method alone. As our focus in this scoping review was on methods rather than results, the CASP tool though informative and useful was perhaps also less robustly applicable compared to systematic reviews.

### Summary

This review has identified substantial variation in both the selection of geriatric syndromes for inclusion in studies and the methods for identifying these in EHRs. We also found that syndromes were not always clearly defined and that the reasons for the choice of syndromes and identification method were often unexplained, despite acknowledged challenges relating to the identification of geriatric syndromes. This should be considered when designing future studies relating to geriatric syndromes. More broadly, establishing clearer guidance regarding how to optimise geriatric syndrome identification may have benefit across both countries with widespread EHR uptake and where this is less embedded.

We recommend future studies describing geriatric syndromes include a minimum pragmatic syndrome set encompassing falls, incontinence, and delirium. These are common across current studies and concepts and are accurately described as syndromes. We also recommend all included syndromes and identification methods are clearly defined and that researchers consider greater use of free text methods to improve yield, particularly for syndromes challenging to code. We strongly support the inclusion of clinical expertise in research teams given the inherent complexities surrounding this topic. These measures may bring greater thematic harmony to a complex topic or improve the potential for cross-study statistical comparability. Finally, a move towards a formal consensus regarding geriatric syndromes may be of overall benefit to both researchers and the older people and healthcare systems that they serve.

## Supplementary Information

Below is the link to the electronic supplementary material.

Supplementary file1 (DOCX 243 KB)
