# Supplementary material for: Extracting geriatric syndromes from electronic health records: a scoping review
Source: Eur Geriatr Med. 2026 Jan 9;17(2):735–49. doi: 10.1007/s41999-025-01388-5 (PMC13109117; doi:10.1007/s41999-025-01388-5)
Supplement: Supplementary file 1 — Supplementary file1 (DOCX 243 KB) [file 41999_2025_1388_MOESM1_ESM.docx]

**Appendices for Extracting Geriatric Syndromes from Electronic Health Records: A Scoping Review**

**Contents**

Appendix 1: PRISMA-Scr checklist

Appendix 2: Full eligibility criteria

Appendix 3: Full search strategy

Appendix 4: CASP checklist

Appendix 5: Descriptions of cognitive disorders in included studies

Appendix 6: References for studies included in scoping review

Appendix 7: Funding sources of included studies

Appendix 8: Acronyms and abbreviations used in main manuscript

**Appendix 1: PRISMA-Scr Checklist**

| **SECTION** | **ITEM** | **PRISMA-ScR CHECKLIST ITEM** | **REPORTED ON PAGE #** |
| --- | --- | --- | --- |
| **TITLE** | | | |
| Title | 1 | Identify the report as a scoping review. | 1 |
| **ABSTRACT** | | | |
| Structured summary | 2 | Provide a structured summary that includes (as applicable): background, objectives, eligibility criteria, sources of evidence, charting methods, results, and conclusions that relate to the review questions and objectives. | 1 |
| **INTRODUCTION** | | | |
| Rationale | 3 | Describe the rationale for the review in the context of what is already known. Explain why the review questions/objectives lend themselves to a scoping review approach. | 2 |
| Objectives | 4 | Provide an explicit statement of the questions and objectives being addressed with reference to their key elements (e.g., population or participants, concepts, and context) or other relevant key elements used to conceptualize the review questions and/or objectives. | 3 |
| **METHODS** | | | |
| Protocol and registration | 5 | Indicate whether a review protocol exists; state if and where it can be accessed (e.g., a Web address); and if available, provide registration information, including the registration number. | 3 |
| Eligibility criteria | 6 | Specify characteristics of the sources of evidence used as eligibility criteria (e.g., years considered, language, and publication status), and provide a rationale. | 3 |
| Information sources* | 7 | Describe all information sources in the search (e.g., databases with dates of coverage and contact with authors to identify additional sources), as well as the date the most recent search was executed. | 3 |
| Search | 8 | Present the full electronic search strategy for at least 1 database, including any limits used, such that it could be repeated. | 3 |
| Selection of sources of evidence† | 9 | State the process for selecting sources of evidence (i.e., screening and eligibility) included in the scoping review. | 3 |
| Data charting process‡ | 10 | Describe the methods of charting data from the included sources of evidence (e.g., calibrated forms or forms that have been tested by the team before their use, and whether data charting was done independently or in duplicate) and any processes for obtaining and confirming data from investigators. | 3 |
| Data items | 11 | List and define all variables for which data were sought and any assumptions and simplifications made. | 3 |
| Critical appraisal of individual sources of evidence§ | 12 | If done, provide a rationale for conducting a critical appraisal of included sources of evidence; describe the methods used and how this information was used in any data synthesis (if appropriate). | 3 |
| Synthesis of results | 13 | Describe the methods of handling and summarizing the data that were charted. | 5 |
| **RESULTS** | | | |
| Selection of sources of evidence | 14 | Give numbers of sources of evidence screened, assessed for eligibility, and included in the review, with reasons for exclusions at each stage, ideally using a flow diagram. | 4 |
| Characteristics of sources of evidence | 15 | For each source of evidence, present characteristics for which data were charted and provide the citations. | 6-9 |
| Critical appraisal within sources of evidence | 16 | If done, present data on critical appraisal of included sources of evidence (see item 12). | NA |
| Results of individual sources of evidence | 17 | For each included source of evidence, present the relevant data that were charted that relate to the review questions and objectives. | 10-12 |
| Synthesis of results | 18 | Summarize and/or present the charting results as they relate to the review questions and objectives. | 10-12 |
| **DISCUSSION** | | | |
| Summary of evidence | 19 | Summarize the main results (including an overview of concepts, themes, and types of evidence available), link to the review questions and objectives, and consider the relevance to key groups. | 12-14 |
| Limitations | 20 | Discuss the limitations of the scoping review process. | 12-14 |
| Conclusions | 21 | Provide a general interpretation of the results with respect to the review questions and objectives, as well as potential implications and/or next steps. | 12-14 |
| **FUNDING** | | | |
| Funding | 22 | Describe sources of funding for the included sources of evidence, as well as sources of funding for the scoping review. Describe the role of the funders of the scoping review. | See Appendix 7 (for included studies). See Funding declaration statement for this review. |

JBI = Joanna Briggs Institute; PRISMA-ScR = Preferred Reporting Items for Systematic reviews and Meta-Analyses extension for Scoping Reviews.

* Where *sources of evidence* (see second footnote) are compiled from, such as bibliographic databases, social media platforms, and Web sites.

† A more inclusive/heterogeneous term used to account for the different types of evidence or data sources (e.g., quantitative and/or qualitative research, expert opinion, and policy documents) that may be eligible in a scoping review as opposed to only studies. This is not to be confused with *information sources* (see first footnote).

‡ The frameworks by Arksey and O’Malley (6) and Levac and colleagues (7) and the JBI guidance (4, 5) refer to the process of data extraction in a scoping review as data charting*.*

§ The process of systematically examining research evidence to assess its validity, results, and relevance before using it to inform a decision. This term is used for items 12 and 19 instead of "risk of bias" (which is more applicable to systematic reviews of interventions) to include and acknowledge the various sources of evidence that may be used in a scoping review (e.g., quantitative and/or qualitative research, expert opinion, and policy document).

*From:* Tricco AC, Lillie E, Zarin W, O'Brien KK, Colquhoun H, Levac D, et al. PRISMA Extension for Scoping Reviews (PRISMAScR): Checklist and Explanation. Ann Intern Med. 2018;169:467–473. [doi: 10.7326/M18-0850](http://annals.org/aim/fullarticle/2700389/prisma-extension-scoping-reviews-prisma-scr-checklist-explanation).

**Appendix 2: Full eligibility criteria**

*Inclusion criteria*

- Include older adults – either a cohort described in this way by study authors or being aged over 65. Where studies include a broader age range, they will be included if older adult outcomes are reported separately. If results are not stratified by age, the study will be excluded.
- Refer to included conditions of interest using descriptors of ‘geriatric syndrome’, ‘frailty syndrome’ or ‘ageing syndrome’
- Include at least two defined geriatric syndromes
- Use electronic health records (EHR), or clearly EHR derived datasets, as the data source for identifying these syndromes. This decision has been made given the contextual differences in medical record structure between nations to optimise relevant study identification.

*Exclusion criteria*

- Studies that do not include adults over 65
- Studies researching conditions that are not clearly described as geriatric syndromes (or frequent synonyms)
- Studies researching frailty status alone: we are excluding this as there has been substantial research regarding both clinical diagnosis of frailty, and diagnosis using algorithms produced from EHRs (such as the eFI), and as there is debate as to whether frailty should be considered a geriatric syndrome in its own right.
- Studies researching dementia alone– these are excluded as studies have already been completed investigating dementia ascertainment from electronic health records, and as dementia diagnoses are considered diseases, rather than syndromes.
- Studies researching a single geriatric condition – these are excluded as the focus of this review regards how studies handle a heterogenous and broadly defined group of conditions, including decisions around selection.
- Studies where it is not clear if the data source was either an EHR, or a derived dataset, unless there is clear evidence that the nation of study had adopted EHRs by at least 95%, at time of data collection.
- Full text not available in English
- Studies using NLP methodology – these were excluded in order to focus on specific methods of identification, and also to enable study findings to be relevant to researchers with different forms and capacities of electronic health records.

**Appendix 3: Full search strategy**

*PubMed*

Search terms: (geriatric syndromes OR frailty syndromes OR ageing syndromes) AND (electronic hospital record OR electronic health record OR electronic medical record OR ehr OR hr OR emr OR hospital record OR medical record) on 24/10/24, updated 31/03/25

*Medline, and CINAHL Plus (via EBSCOhost)*

Search terms: (geriatric syndromes OR frailty syndromes OR ageing syndromes) AND (electronic hospital record OR electronic health record OR electronic medical record OR ehr OR hr OR emr OR hospital record OR medical record) on 24/10/24, updated 31/03/25

*SCOPUS*

Search terms: Search terms: (geriatric syndromes OR frailty syndromes OR ageing syndromes) AND (electronic hospital record OR electronic health record OR electronic medical record OR ehr OR hr OR emr OR hospital record OR medical record)

Date: 4/11/24, updated 31/03/25

**Appendix 4: CASP checklist**

**Appendix 5: Descriptions of cognitive syndromes and identification methods**

***Number in brackets indicates reference number in main article bibliography***

* Oh ES, Fong TG, Hshieh TT, Inouye SK. Delirium in older persons: Advances in diagnosis and treatment. JAMA. 2017;318(12):1161-1174'

** Delirium risk was assessed with three items: memory problems, history of an acute episode of confusion or delirium and help in activities of daily living (ADL). Delirium risk was scored as present if a patient scored ≥ 1 item (out of 3) positive

**Appendix 6: Studies used in scoping review analysis**

1 (38). Oud FMM, Schut MC, Spies PE, van der Zaag-Loonen HJ, de Rooij SE, Abu-Hanna A, et al. Interaction between geriatric syndromes in predicting three months mortality risk. Arch Gerontol Geriatr. 2022 Nov 1;103.

2 (10). Chew J, Chia JQ, Kyaw KK, Fu KJ, Lim C, Chua S, et al. Frailty Screening and Detection of Geriatric Syndromes in Acute Inpatient Care: Impact on Hospital Length of Stay and 30-Day Readmissions. Ann Geriatr Med Res [Internet]. 2023;27(4):315–23. Available from: https://www.scopus.com/inward/record.uri?eid=2-s2.0-85180894507&doi=10.4235%2fagmr.23.0124&partnerID=40&md5=6146cf5ec2f043317fd93470bf2a7431

3 (40). Liang X, Li X, Cheng H, Wei F, Li T, Li Y, et al. Elderly patients with dysphagia in the intensive care unit: Association between malnutrition and delirium. Nurs Crit Care [Internet]. 2024; Available from: https://www.scopus.com/inward/record.uri?eid=2-s2.0-85201282899&doi=10.1111%2fnicc.13136&partnerID=40&md5=b9b394fd52481293d6303167672689df

4 (36). Lozano-Montoya I, Quezada-Feijoo M, Jaramillo-Hidalgo J, Garmendia-Prieto B, Lisette-Carrillo P, Gómez-Pavón FJ. Mortality risk factors in a Spanish cohort of oldest-old patients hospitalized with COVID-19 in an acute geriatric unit: the OCTA-COVID study. Eur Geriatr Med [Internet]. 2021;12(6):1169–80. Available from: https://login.eux.idm.oclc.org/login?url=https://search.ebscohost.com/login.aspx?direct=true&db=cmedm&AN=34287813&site=ehost-live

5 (39). Szklarzewska S, Vande Walle J, De Breucker S, Schoevaerdts D. A comparison of clinical characteristics between old and oldest-old patients hospitalised for SARS-COV2. Acta Clin Belg [Internet]. 2023;78(3):192–9. Available from: https://login.eux.idm.oclc.org/login?url=https://search.ebscohost.com/login.aspx?direct=true&db=cmedm&AN=35894148&site=ehost-live

6 (44). Faitna P, Bottle A, Klaber B, Aylin PP. Has multimorbidity and frailty in adult hospital admissions changed over the last 15 years? A retrospective study of 107 million admissions in England. BMC Med [Internet]. 2024;22(1):369. Available from: https://login.eux.idm.oclc.org/login?url=https://search.ebscohost.com/login.aspx?direct=true&db=cmedm&AN=39256751&site=ehost-live

7(34). S DB, Herzog G, Pepersack T. Could geriatric characteristics explain the under-prescription of anticoagulation therapy for older patients admitted with atrial fibrillation? Drugs Aging [Internet]. 2010;27(10):807–13. Available from: https://login.eux.idm.oclc.org/login?url=https://search.ebscohost.com/login.aspx?direct=true&db=jlh&AN=104958643&site=ehost-live

8 (43). Rönneikkö JK, Jämsen ER, Mäkelä M, Finne-Soveri H, Valvanne JN. Reasons for home care clients’ unplanned Hospital admissions and their associations with patient characteristics. Arch Gerontol Geriatr [Internet]. 2018;78:114–26. Available from: https://login.eux.idm.oclc.org/login?url=https://search.ebscohost.com/login.aspx?direct=true&db=jlh&AN=131090848&site=ehost-live

9 (37). Ohuabunwa U, Afolabi P, Tom‐Aba D, Fluker S. Clinical presentation of COVID‐19 and association with outcomes among hospitalized older adults. J Am Geriatr Soc [Internet]. 2023;71(2):599–608. Available from: https://login.eux.idm.oclc.org/login?url=https://search.ebscohost.com/login.aspx?direct=true&db=jlh&AN=161967397&site=ehost-live

10 (35). Lin RJ, Hilden PD, Elko TA, Dahi PB, Shahrokni A, Jakubowski AA, et al. Burden and impact of multifactorial geriatric syndromes in allogeneic hematopoietic cell transplantation for older adults. Blood Adv [Internet]. 2019;3(1):12–20. Available from: https://login.eux.idm.oclc.org/login?url=https://search.ebscohost.com/login.aspx?direct=true&db=cmedm&AN=30606722&site=ehost-live

11 (41). Soong J, Poots AJ, Scott S, Donald K, Woodcock T, Lovett D, et al. Quantifying the prevalence of frailty in English hospitals. BMJ Open. 2015;5(10):e008456-.

12 (42). Melzer D, Tavakoly B, Winder RE, Masoli JAH, Henley WE, Ble A, et al. Much more medicine for the oldest old: trends in UK electronic clinical records. Age Ageing [Internet]. 2015;44(1):46–53. Available from: <https://login.eux.idm.oclc.org/login?url=https://search.ebscohost.com/login.aspx?direct=true&db=jlh&AN=103869455&site=ehost-live>

| First Author | Year | Funding Information Acknowledged in Studies |
| --- | --- | --- |
| Chew | 2023 | None declared |
| De Bruecker | 2024 | None declared |
| Faitna | 2024 | This study was funded by the National Institute of Health Research (NIHR) Imperial Patient Safety Research Collaboration (PSTRC), the Imperial NIHR Biomedical Research Centre (BRC) and the Imperial College London President’s PhD Scholarship. NIHR PSTRC and NIHR BRC are partnerships between the Imperial College Healthcare NHS Trust and Imperial College London. This report is independent research supported by the National Institute for Health and Care Research Applied Research Collaboration Northwest London. The views expressed in this publication are those of the author(s) and not necessarily those of the National Institute for Health Research or the Department of Health and Social Care. The open access fee was paid from the Imperial College London Open Access Fund. |
| Liang | 2024 | This study was supported by the Clinical Frontier Technology Program of the First Affiliated Hospital of Jinan University, China (No. JNU1AF-CFTP-2022-a01235), the Science and Technology Projects in Guangzhou, China (Nos 202201020054 and 2023A03J1032) and the Hospital Research Projects in Guangzhou, China (No. 2022106). |
| Lin | 2019 | This work was supported in part by grants from the National Institutes of Health, National Cancer Institute (P01 CA23766) and Cancer Center Support (P30 CA008748) (to Memorial Sloan Kettering Cancer Center). R.J.L. was supported by the New York State Empire Clinical Research Investigator Program and the Elsa U. Pardee Foundation for Cancer Research.  The content is solely the responsibility of the authors and does not necessarily represent the official views of the National Institutes of Health. |
| Lozano-Montoya | 2021 | ‘The OCTA-COVID authors have not declared a specific grant for this work’ |
| Melzer | 2015 | Study supported by Age UK (registered charity 1128267). Author funding includes NIHR School for Public Health Research, NIHR Clinical Fellowship Award, NIHR Collaboration for Leadership in Applied Health Research and Care for the South West Peninsula. Financial sponsors played no role in the design, execution, analysis and interpretation of data, or writing of the study. |
| Ohuabunwa | 2023 | None declared |
| Oud | 2022 | None declared |
| Rönneikkö | 2018 | This research did not receive any specific grant from funding agencies in the public, commercial, or not-for-profit sectors. |
| Soong | 2015 | None declared |
| Szklarzewska | 2023 | Not reported |

**Appendix 7: Funding declarations of included studies**

**Appendix 8: Acronyms and abbreviations**

- GS: Geriatric syndromes
- EHR: electronic medical record
- BGS: British Geriatric Society
- AGS: American Geriatric Society
- APGS: Asian-Pacific Geriatric Society
- VMS: Dutch safety management screening tool for frail older patients
- HES: Hospital episode statistics (dataset)
- CPRD: Clinical Research Practice Datalink
- THL: Finnish Institute for Health and Welfare
- FHDR: Finnish Hospital Discharge Register
- MIMIC-IV: Medical Information Mart for Intensive Care IV
